# Unfavorable impact of cancer cachexia on activity of daily living and need for inpatient care in elderly patients with advanced non-small-cell lung cancer in Japan: a prospective longitudinal observational study

**DOI:** 10.1186/s12885-017-3795-2

**Published:** 2017-11-28

**Authors:** Tateaki Naito, Taro Okayama, Takashi Aoyama, Takuya Ohashi, Yoshiyuki Masuda, Madoka Kimura, Hitomi Shiozaki, Haruyasu Murakami, Hirotsugu Kenmotsu, Tetsuhiko Taira, Akira Ono, Kazushige Wakuda, Hisao Imai, Takuya Oyakawa, Takeshi Ishii, Shota Omori, Kazuhisa Nakashima, Masahiro Endo, Katsuhiro Omae, Keita Mori, Nobuyuki Yamamoto, Akira Tanuma, Toshiaki Takahashi

**Affiliations:** 1 Division of Thoracic Oncology, Shizuoka Cancer Center, 1007, Shimonagakubo, Nagaizumi-cho, Sunto-gun, Shizuoka, 411-8777 Japan; 2 Division of Rehabilitation Medicine, Shizuoka Cancer Center, 1007, Shimonagakubo, Nagaizumi-cho, Sunto-gun, Shizuoka, 411-8777 Japan; 3 Division of Nutrition, Shizuoka Cancer Center, 1007, Shimonagakubo, Nagaizumi-cho, Sunto-gun, Shizuoka, 411-8777 Japan; 4 Division of Physical Medicine and Rehabilitation, Shizuoka General Hospital, 4-27-1 Kita Ando Aoi-ku, Shizuoka, 420-8527 Japan; 5 Department of Clinical Oncology, Osaka Medical Center for Cancer and Cardiovascular Diseases, 1-3-3 Nakamichi, Tosei-ku, Osaka, 537-8511 Japan; 6 Division of Respiratory Medicine, Gunma Prefectural Cancer Center, 617-1 Takabayashi-nishi-machi, Ohta-shi, Gunma, 373-8550 Japan; 7 Division of Cardiology, Shizuoka Cancer Center, 1007, Shimonagakubo, Nagaizumi-cho, Sunto-gun, Shizuoka, 411-8777 Japan; 8 Division of Diagnostic Radiology, Shizuoka Cancer Center, 1007, Shimonagakubo, Nagaizumi-cho, Sunto-gun, Shizuoka, 411-8777 Japan; 9 Clinical Research Center, Shizuoka Cancer Center, 1007, Shimonagakubo, Nagaizumi-cho, Sunto-gun, Shizuoka, 411-8777 Japan; 10 Third Department of Internal Medicine, Wakayama Medical University, 811-1, Kimiidera, Wakayama, 641-8509 Japan

**Keywords:** Non-small-cell lung cancer, Elderly, Cancer cachexia, Activity of daily living, Length of hospital stay, Medical cost

## Abstract

**Background:**

Cancer cachexia in elderly patients may substantially impact physical function and medical dependency. The aim of this study was to estimate the impact of cachexia on activity of daily living (ADL), length of hospital stay, and inpatient medical costs among elderly patients with advanced non-small-cell lung cancer (NSCLC) receiving chemotherapy.

**Methods:**

Thirty patients aged ≥70 years with advanced NSCLC (stage III-IV) scheduled to receive first-line chemotherapy were prospectively enrolled between January 2013 and November 2014. ADL was assessed using the Barthel index. The disability-free survival time (DFS) was calculated as the time between the date of study entry and the date of onset of a disabling event, which was defined as a 10-point decrease in the Barthel index from that at baseline. The mean cumulative function of the length of hospital stay and inpatient medical costs (¥, Japanese yen) was calculated.

**Results:**

The study patients comprised 11 women and 19 men, with a median age of 74 (range, 70–82) years. Cachexia was diagnosed in 19 (63%) patients. Cachectic patients had a shorter DFS (7.5 vs. 17.1 months, *p* < 0.05). During the first year from study entry, cachectic patients had longer cumulative lengths of hospital stay (80.7 vs. 38.5 days/person, *p* < 0.05), more frequent unplanned hospital visits or hospitalizations (4.2 vs. 1.7 times/person, *p* < 0.05), and higher inpatient medical costs (¥3.5 vs. ¥2.1 million/person, *p* < 0.05) than non-cachectic patients.

**Conclusions:**

Elderly NSCLC patients with cachexia showed higher risks for disability, prolonged hospitalizations, and higher inpatient medical costs while receiving chemotherapy than patients without cachexia. Our results might indicate that there is a potential need for an early intervention to minimize progression to or development of cachexia, improve functional prognosis, and reduce healthcare resource burden in this population.

**Trial registration:**

Trial registration number: UMIN000009768. Name of registry: UMIN (University hospital Medical Information Network). Date of registration: 14 January 2013. Date of enrolment of the first participant to the trial: 23 January 2013.

## Background

The number of elderly people living with advanced lung cancer is increasing worldwide, owing to the aging population and progress in cancer treatments [1]. In Japan, 65% of lung cancer morbidity cases and 73% of lung cancer-related deaths were attributed to elderly individuals aged ≥70 years in 2012 [2]. Patients with lung cancer, especially the elderly population, often develop dependency in activities of daily living (ADLs) during treatment [3, 4]. In addition, the financial burden of elderly lung cancer patients is growing. In Japan, about half (52%, ¥222 billion) of the annual national costs for tracheal, bronchus, and lung cancers are attributed to elderly individuals aged ≥70 years, and the majority of these funds (75%, ¥166 billion) are used for their inpatient care [5]. Thus, the socioeconomic impact of elderly lung cancer patients is serious and cannot be ignored.

Cancer cachexia is a multifactorial syndrome characterized by a significant reduction in body weight associated with reduced muscle and adipose tissue mass [6]. Cancer cachexia is frequently observed in advanced lung cancer patients not only in the terminal phase of the disease, but also in the early phase of cancer diagnosis [6, 7]. We have previously reported that approximately half of all patients with newly diagnosed advanced non-small-cell lung cancer (NSCLC) had cachexia and skeletal muscle mass depletion at the time of diagnosis. The skeletal muscle mass further decreases during subsequent chemotherapies along with loss of physical function [8]. Standard treatment for cancer cachexia has not been established. However, a number of pharmacological agents [9–11] and multimodal care approaches [12] are currently being assessed in clinical trials. Patients with cancer cachexia have poor physical function [13] and are at high risk for disabilities, prolonged hospitalizations, and in-hospital death [14]. As a result, patients with cancer cachexia are less likely to tolerate cancer treatment [15] and have poorer quality of life and prognosis [8, 16]. Recently, the presence of cancer cachexia was reported to have a substantial socioeconomic impact on cancer care [14, 17]. Thus, effective management of cancer cachexia may decrease medical dependency and the need for inpatient care. However, there is currently limited information about the socioeconomic impact of cachexia in elderly patients living with advanced NSCLC who are receiving palliative chemotherapy.

Accordingly, this study aimed to estimate the impact of cachexia on ADL, length of hospital stay, and inpatient medical costs among elderly patients with advanced NSCLC receiving chemotherapy.

## Methods

### Patient selection

This prospective longitudinal observational study was designed to estimate the impact of cachexia on ADL, length of hospital stay, and inpatient medical costs among elderly patients with advanced NSCLC receiving chemotherapy. The study was performed at the Shizuoka Cancer Center, Japan, from January 2013 to April 2016. The Shizuoka Cancer Center is a 615-bed prefectural hospital designated as an advanced treatment hospital by the Japanese Ministry of Health, Labour and Welfare. The eligibility criteria were as follows: (1) histologically and/or cytologically proven stage III or IV NSCLC including postoperative recurrence; (2) age ≥ 70 years, with scheduled first-line systemic chemotherapy; (3) no previous systemic chemotherapy or thoracic radiotherapy (adjuvant chemotherapy was not counted as prior chemotherapy); (4) Eastern Cooperative Oncology Group performance status of 0–2; (5) ability to ambulate, read, and respond to questions without assistance; and (6) expected survival of >12 weeks. Patients were excluded if they had a severe psychiatric disorder, active infectious disease, unstable cardiac disease, or untreated symptomatic brain or bone metastases that prevented safe assessment.

All patients provided written informed consent. The study was approved by the institutional review board and registered on the clinical trials site of the University Hospital Medical Information Network Clinical Trials Registry in Japan (registration number: UMIN000009768).

### Patient enrollment and timing of data collection

The first patient was enrolled on January 23, 2013, and the last on November 7, 2013. The study period for each patient was defined as the time between the date of study entry to the date of the last visit or the cutoff date (April 30, 2016). Baseline study assessments were performed by the attending physicians, physiotherapists, and national registered dietitians during the time between study entry and initiation of the first chemotherapy session.

### Patient assessment

Body weight (kg) was measured to the nearest 0.1 kg and the body mass index (BMI; kg/m^2^) was subsequently calculated. The registered dietitians (T.A. and H.S.) assessed nutritional status using the full version of the Mini Nutritional Assessment (full MNA®) [18]. The incremental shuttle-walking test was conducted according to the recent guidelines [19] and original protocol described by Singh et al. [20]. The 10-m course was established in the corridor of our hospital. Walking speed was dictated by a timed signal played on a CD recorder provided by the manufacturer (Japanese version, produced by the Graduate School of Biomedical Sciences, Nagasaki University, Japan, 2000). All patients were subjected to the test once under standardized conditions and were carefully observed during the test, so that they would not exceed their exercise limit. The maximal walking distance was described as incremental shuttle-walking distance. Hand-grip strength was measured using a grip strength dynamometer (GRIP-D, Takei Scientific Instruments Co., LTD, Niigata, Japan). One trial was performed for each hand, and the result from the strongest hand was used for the analysis. Lumbar skeletal muscle mass was measured by analyzing electronically stored computed tomography (CT) images using SYNAPSE VINCENT version 3 (FUJIFILM Medical Systems, Japan). The CT images were obtained with or without contrast enhancement at 5-mm slice thickness. The third lumbar vertebra (L3) was chosen as the standard landmark, and 2 consecutive CT images extending from L3 to the iliac crest were chosen to measure the cross-sectional area of the skeletal muscle that was identified based on Hounsfield unit thresholds of −29 to +150. The sum of the cross-sectional areas (cm^2^) of the muscles in the L3 region was computed for each image. The mean value of 2 images was normalized for height in meters squared and reported as the lumbar skeletal muscle index (cm^2^/m^2^) [21]. The disease stage was determined according to the TNM classification, and the best response to chemotherapy was evaluated according to the Response Evaluation Criteria in Solid Tumors.

### Diagnosis of muscle depletion, malnutrition, and cancer cachexia

Muscle depletion was defined based on lumbar skeletal muscle index cutoffs of <43.0 cm^2^/m^2^ for men with a BMI <25.0 kg/m^2^, <53.0 cm^2^/m^2^ for men with a BMI ≥25.0 kg/m^2^, and <41.0 cm^2^/m^2^ for women [22]. Malnutrition or at risk of malnutrition was defined based on a full MNA® score < 17 points [23]. Cancer cachexia was defined as unintentional weight loss of >5% during the preceding 6 months or >2% in patients with a BMI <20 kg/m^2^, or the presence of muscle depletion according to the consensus criteria [6]. The patient’s weight 6 months before study entry was obtained by interviewing the patient and their family members at study entry.

### Assessment of activity of daily living

For the assessment of ADL, the Barthel index was estimated by the attending physician or physiotherapists at each hospital visit. The disability-free survival (DFS) duration was calculated as the time between study enrollment and the date of onset of the disabling event. A disabling event was defined as a decrease in the Barthel index from the baseline value by >10 points. The event was confirmed as a true event if the condition persisted for >2 weeks from the initial report. In confirmed events, the dates of the initial reports were used as the event dates in the analysis.

### Assessment of healthcare resource utilization

The medical claims data, including the numbers of outpatient visits and hospitalizations, lengths of hospital stay, healthcare resource utilization items, and medical costs, were obtained from the electronic medical records of our hospital. For patients who received medical care at another hospital, medical claims data was obtained through the institutional coordination office of local clinics and hospitals. Inpatient medical costs were estimated by certified medical accountants. In this study, the medical costs refer to the actual revenue that the hospital was paid from the health insurance funds of the Japanese health care system. Medical costs for home care were not included. In the healthcare utilization analysis, visits (or hospitalizations) for supportive care were defined as all visits (or hospitalizations) that involved physician medical examinations, except for visits (or hospitalizations) for chemotherapy or radiotherapy. Outpatient visits for regular radiological/blood tests without a physician examination or visits for non-medical reasons were not included.

### Statistical analysis

The overall survival (OS) and DFS rates were estimated using the Kaplan-Meier method. OS was censored at the date of the last visit for patients whose deaths could not be confirmed. DFS was censored at the date of the last visit for patients whose disabling event could not be confirmed. To compare categorical variables, chi-square or Fisher’s exact tests were used. Continuous measures were compared using the Wilcoxon rank-sum test. For all analyses, *p*-values <0.05 were considered significant. We used a mean cumulative function for recurrent event analysis [24] of the cumulative length of hospital stay and medical costs related to cancer care, as previously described [25, 26]. Exploratory subset analyses for patients without epidermal growth factor receptor (EGFR) gene mutation were performed. All statistical analyses were performed using JMP version 12.0 for Windows (SAS Institute Inc., USA).

## Results

### Patients

Thirty-one patients were screened and 30 patients were enrolled into this study, with a median age of 74 years (range, 70–82 years). Seven patients (23.3%) had activating EGFR gene mutation. Cancer cachexia was diagnosed in 19 (63.3%) patients (Table 1). Cachectic patients were older (76 vs. 73 years, *p* < 0.05), had a larger weight loss in the past 6 months (−9.4 vs. −0.1%, *p* < 0.05), and had a higher incidence of malnutrition or were more frequently at risk for malnutrition (73.7 vs. 27.3%, *p* < 0.05). Cachectic men had poorer physical function than non-cachectic men in regard to the incremental shuttle-walking distance (283.4 vs. 413.8 m, *p* < 0.05) and hand-grip strength (29.5 vs. 39.3 kg, *p* < 0.05). There was no statistical difference in physical function between cachectic and non-cachectic women.

**Table 1:** Baseline patient characteristics

Variables	All *N* = 30	Cachexia *N* = 19	Non-cachexia *N* = 11	*p*-value*
Age, median (range)	74 (70–82)	76 (70–82)	73 (70–80)	<0.05
Gender (Women:Men)	11:19	8:11	3:8	NS
ECOG-PS, n (%)				
0	11 (36.7)	5 (26.3)	6 (54.6)	NS
1	18 (60.0)	13 (68.4)	5 (45.5)	
2	1 (3.3)	1 (5.3)	0 (0.0)	
Stage, n (%)				
IIIA or IIIB	1 (3.3)	1 (5.3)	0 (0.0)	NS
IV or postoperative recurrence	29 (96.7)	18 (94.7)	11 (100.0)	
Tumor Histology, n (%)				
Adenocarcinoma	21 (70.0)	13 (68.4)	8 (72.7)	NS
Other non-small-cell lung cancer	9 (30.0)	6 (31.6)	3 (27.3)	
EGFR gene				
Mutant	7 (23.3)	5 (26.3)	2 (18.2)	NS
Wild type or unknown	23 (76.7)	14 (73.7)	9 (81.8)	
Treatment, n (%)				
Cytotoxic regimen	24 (80.0)	15 (79.0)	9 (81.8)	NS
Targeted regimen	6 (20.0)	4 (21.0)	2 (18.2)	
Nutrition				
BMI (kg/m^2^)	21.1 ± 3.4	20.4 ± 2.8	22.2 ± 1.2	NS
% weight change in prior 6 months (%, mean ± SD)	−6.0 ± 6.4	−9.4 ± 5.5	−0.1 ± 2.2	<0.05
Malnutrition or at risk of malnutrition^a^, n (%)	17 (56.7)	14 (73.7)	3 (27.3)	<0.05
Skeletal muscle depletion^b^	20 (66.7)	13 (68.4)	7 (63.6)	NS
Lumbar skeletal muscle index (cm^2^/m^2^)				
Women	35.4 ± 4.1	34.1 ± 3.9	39.0 ± 2.2	NS
Men	44.5 ± 7.6	42.3 ± 6.1	47.6 ± 8.8	NS
Physical capacity				
Incremental shuttle walking distance (m)				
Women	304.5 ± 99.2	338.8 ± 90.5	213.3 ± 58.6	NS
Men	338.4 ± 143.0	283.6 ± 146.5	413.8 ± 103.5	<0.05
Hand-grip-strength in dominant side (kg)				
Women	21.7 ± 4.1	22.4 ± 4.5	20.0 ± 2.7	NS
Men	33.9 ± 7.1	29.5 ± 4.3	39.3 ± 6.0	<0.05

*ECOG-PS* Eastern cooperative oncology group performance status, *EGFR* epidermal growth factor receptor, *BMI* body-mass-index, *NS* not significant, *SD* standard deviation

* Significant difference (*P* < 0.05) tested by Chi-square test, Fisher exact test, or Wilcoxon test

^a^Malnutrition or at risk of malnutrition was defined based on the full version of Mini nutritional assessment score < 17 points. ^b^skeletal muscle depletion was defined as lumbar skeletal muscle mass index of <43.0 cm^2^/m^2^ for men with a BMI <25.0 kg/m^2^, <53.0 cm^2^/m^2^ for men with a BMI ≥25.0, and <41.0 cm^2^/m^2^ in women

### Cancer treatment during the study period

**Fig. 1:**
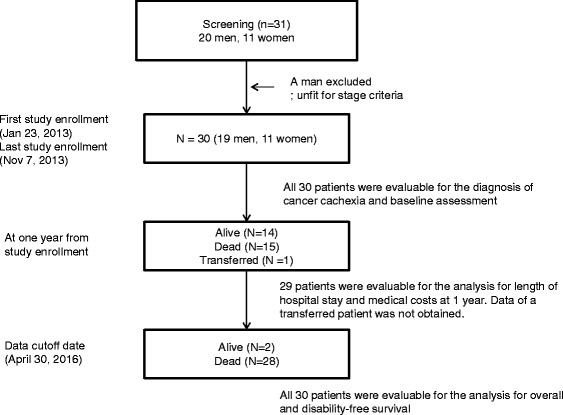
Patient flow chart

All patients received first-line chemotherapy within 1 week after the baseline assessment. All patients initially received a standard dose of chemotherapy with a standard schedule. The chemotherapy regimens included single-agent chemotherapy (docetaxel or vinorelbine) in 10 patients, platinum-based chemotherapy (carboplatin + paclitaxel, or cisplatin + pemetrexed, gemcitabine, or vinorelbine) in 14, and gefitinib in 6 patients with epidermal growth factor receptor gene mutations. An objective tumor response was seen in 12 patients (40.0%). There was no statistical difference in the response rate between cachectic and non-cachectic patients (42.1% vs. 36.4%, *p* = 0.75). During the study period (January 23, 2013 to April 30, 2016), 18 patients (60.0%) received second or higher lines of chemotherapy, including docetaxel, erlotinib, pemetrexed, gemcitabine, carboplatin + pemetrexed, S-1, or investigational drugs. There was no statistical difference in the proportion of patients receiving second or higher lines of chemotherapy between cachectic and non-cachectic patients. Eighteen patients (60.0%) received palliative radiotherapy during the study period, including cranial radiation (*n* = 10, 33.3%), bone radiation (*n* = 6, 20.0%), and thoracic radiation (*n* = 2, 6.7%). None of our patients received immunotherapy or molecular targeted treatment other than gefitinib or erlotinib during the study period. One patient was transferred to another hospital for personal reasons 19.4 weeks after study entry and continued chemotherapy. Three patients were transferred to another hospital for palliative care. Three patients received gamma knife surgery at another hospital during the study period. A total of 29 patients were eligible for the analysis of length of hospital stay and medical costs (Fig. 1). None of our patients received anti-cachexia treatment such as megestrol acetate, eicosapentaenoic acid, or multimodal intervention specific for cancer cachexia.

### Follow-up period and overall survival

**Fig. 2:**
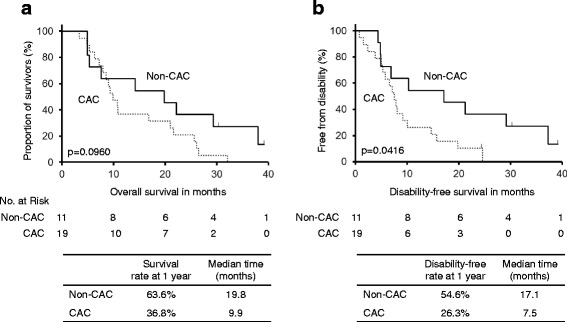
Overall and disability-free survival curves. **a** Kaplan-Meier curve of overall survival. **b** Kaplan-Meier curve of disability-free survival. *P*-values were calculated using log-rank tests. Disabling events were defined as a decrease in the Barthel index from the baseline value by >10 points. For patients whose disabling event could not be confirmed, it was censored at the date of the last visit. CAC, cancer cachexia

Among the 30 patients, 28 (93.3%) died at the cutoff date. The median follow-up period was 10.7 (95% confidence interval, 7.9–21.6) months. There was no significant difference in OS between cachectic and non-cachectic patients (*p* = 0.0960, Fig. 2a). In the exploratory analysis for patients without EGFR mutation, there was also no significant difference in OS between cachectic and non-cachectic patients (*p* = 0.2055).

### Disabling events and disability-free survival

**Fig. 3:**
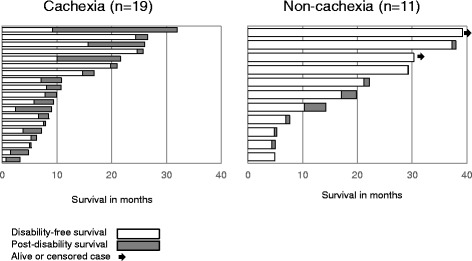
Event plots for disability-free and post-disability survival. The bars represent the duration in months of disability-free (white) and post-disability (gray) survival for each of the 19 cachectic patients and 11 non-cachectic patients. The arrows represent the patients alive or censored at the data cutoff date (April 30, 2016). Disabling events were defined as a decrease in the Barthel index from the baseline value by >10 points

Among the 30 patients, 27 (90.0%) were disabled at the cutoff date. Disabling events often affected multiple ADLs simultaneously. Frequently observed combinations of initial disabling events per the Barthel index included stair climbing (27 events, 100%), morbidity (26 events, 96.3%), bathing (24 events, 88.9%), toilet use (15 events, 55.6%), and transfer (11 events, 40.7%). Cachectic patients at baseline had a significantly shorter DFS than non-cachectic patients (7.5 vs. 17.1 months, *p* < 0.05, Fig. 2b). Additionally, cachectic patients had a longer post-disability survival than non-cachectic patients (2.5 vs. 0.7 months, *p* < 0.05, Fig. 3). In the exploratory analysis for patients without EGFR mutation, cachectic patients tended to have shorter DFS (6.8 vs. 10.3 months, *p* = 0.1078) and longer post-disability survival (2.6 vs. 0.6 months, *p* = 0.0541) than non-cachectic patients without statistical significance.

### Healthcare resource utilization

During the first year since study entry, we recorded 525 outpatient visits and 144 hospitalizations for the 29 patients. There were 42 unplanned outpatient visits (8.0% of all outpatient visits), including 14 visits (2.7%) to the emergency room. The reasons for the unplanned visits included anorexia or dehydration (17 visits, 41%), infection or febrile disease (9 visits, 21%), constipation or diarrhea (5 visits, 12%), respiratory symptoms (5 visits, 12%), and others (6 visits, 14%). There were 30 emergency hospitalizations (20.8% of all hospitalizations). The reasons for emergency hospitalizations included anorexia or dehydration (7 hospitalizations, 23%), respiratory symptoms (6 hospitalizations, 20%), infection or febrile disease (5 hospitalizations, 17%), urgent radiotherapy (4 hospitalizations, 13%), end-of-life care (3 hospitalizations, 10%), and others (5 hospitalizations, 17%). Hospitalization for non-medical reasons (e.g. social hospitalization) was not observed.

### Length of hospital stay and medical cost for cachectic patients

**Fig. 4:**
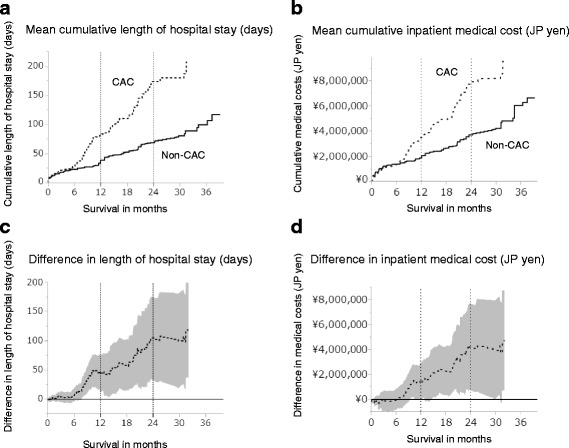
Difference between cachectic and non-cachectic patients in terms of cumulative hospital days and medical costs. Curves of mean cumulative functions for length of hospital stay (**a**) and medical costs (**b**) in cachectic (dotted-line) and non-cachectic (solid-line) patients. Curves of mean differences in the length of hospital stay (**c**) and medical costs (**d**). The colored area represents the 95% confidence interval of the mean difference. CAC, cancer cachexia

In the comparison of socioeconomic parameters during the first year of study entry, cachectic patients had a longer cumulative length of hospital stay (80.7 vs. 38.5 days/person, *p* < 0.05, Table 2), more frequent unplanned outpatient visits or emergency hospitalizations (4.2 vs. 1.7 times/person, *p* < 0.05, Table 2), and higher cumulative medical costs (¥5.0 vs. ¥3.3 million/person, *p* < 0.05, Table 2). In the exploratory analysis for patients without EGFR mutation, cachectic patients had a longer cumulative length of hospital stay (109.3 vs. 45.9 days/person, *p* < 0.05), more frequent unplanned outpatient visits or emergency hospitalizations (5.5 vs. 2.0 times/person, *p* < 0.05), and higher cumulative medical costs (¥5.3 vs. ¥3.3 million/person, *p* < 0.05). The differences in medical costs between the two groups were mainly attributed to the inpatient care (¥3.5 vs. 2.1 million, *p* < 0.05) and supportive care (¥1.2 vs. 0.4 million, *p* < 0.05), while the costs for outpatient care (¥1.5 vs. 1.3 million) and anticancer treatments, including radiotherapy (¥0.5 vs. 0.2 million) and chemotherapy (¥2.0 vs. 2.2 million), were similar between the groups (Table 2). The curves of cumulative hospital days (Fig. 4a) and inpatient medical costs (Fig. 4b) in cachectic and non-cachectic patients separated at 6 months and continued to diverge over the available follow-up period, with the length of hospitalization being 42.2 and 104.3 days (Fig. 4c) and in inpatient medical costs ¥1.5 and ¥4.2 million (Fig. 4d) at 12 and 24 months, respectively.

**Table 2:** Differences in socioeconomic parameters in the first year of cancer treatment

Socioeconomic parameters for the first year	Cachexia	Non-Cachexia	*p*-value
Cumulative no. of hospital stay (days per person)	80.7 ± 13.7	38.5 ± 8.7	<0.05
Cumulative no. of unplanned visits or emergency hospitalizations (times per person)	4.2 ± 1.0	1.7 ± 0.5	<0.05
Cumulative medical costs (×10^6^ JP yen per person)			
Total	5.0 ± 0.4	3.3 ± 0.5	<0.05
Outpatient care	1.5 ± 0.3	1.3 ± 0.3	NS
Inpatient care	3.5 ± 0.6	2.1 ± 0.5	<0.05
Cumulative costs for resource utilization (×10^6^ JP yen per person)			
Chemotherapy	2.0 ± 0.3	2.2 ± 0.3	NS
Radiotherapy	0.5 ± 0.1	0.2 ± 0.1	NS
Supportive care	1.2 ± 0.3	0.4 ± 0.1	<0.05

## Discussion

To our knowledge, this is the first prospective longitudinal observational study to evaluate the impact of cachexia on functional prognosis and socioeconomic parameters in elderly patients with advanced NSCLC. First, we found that cachectic patients tended to have lower muscle mass, muscle strength, and walking capacity than non-cachectic patients, especially the men. Second, in regards to functional prognosis, cachectic patients were found to be disabled earlier and have a longer duration of disability in their life time than non-cachectic patients. Third, from a socioeconomic view, cachectic patients required longer hospital stays and higher inpatient medical costs than non-cachectic patients, mainly due to the increased needs for supportive care rather than anti-cancer treatment.

Cancer cachexia is a hypercatabolic condition that cannot be simply reversed by energy supplementation [6]. A previous study reported that two-thirds of incurable chemotherapy-naïve NSCLC patients experienced weight loss at the time of diagnosis [7] and a majority of them met the recent diagnostic criteria for cancer cachexia [6], as reported in our previous study [8]. High incidences of malnutrition and sarcopenia have been reported in advanced lung cancer patients [22, 27]. Regarding physical function in patients with advanced NSCLC, cachectic patients had worse physical function at baseline and the rate of decline in physical function was more rapid than that in non-cachectic patients during the course of cancer treatment [13].

Arthur et al. [14, 28] recently reported that the presence of cachexia was strongly associated with higher risks for major loss of function (i.e. disability) and increased inpatient costs, not only in the general population but also in cancer patients. Among the 5 most common cancer types associated with cachexia, namely lung cancer, pancreatic cancer, esophageal cancer, stomach cancer, and Kaposi’s sarcoma, cachectic lung cancer patients are at the highest risk for major loss of function. Consistently, our study showed that cachectic patients were more likely to be disabled during their course of cancer treatment. The possible reasons for the increased susceptibility to disabling events in cachectic patients might include older age and higher incidence of malnutrition and muscle depletion in this subset of patients, as these conditions have been reported to be associated with functional impairment and disability [29–31].

There is currently limited information on the socioeconomic impact of cancer cachexia, including medical costs and use of healthcare resources [17]. Cachectic lung cancer patients have been reported to require longer lengths of hospital stay, which in turn leads to a higher financial burden [14]. Our data fully support this previous finding. In addition, our analysis provided more detailed information about the breakdown of healthcare resource utilization items. Most differences in medical costs between cachectic and non-cachectic patients could be attributed to the requirements for inpatient palliative care. Conversely, there were few differences in the costs associated with out-patient care and active cancer treatments between the groups.

Our study has several limitations. First, this study involved a small sample size that included only Japanese patients treated at a single institution. Second, our study population was heterogeneous in regard to the treatment regimens received. Additionally, there was a difference in age between the cachectic and non-cachectic groups. However, exploratory analyses for patients without EGFR mutation showed small impact on the results and age also had little impact on the comparison of endpoints (data not shown). Third, this study lacks a measurement of quality of life that may be important to estimate the net impact of cancer cachexia in comparison with the medical costs. Finally, the health insurance system in Japan differs from that of other countries or regions. Moreover, the medical environment and standard of care are rapidly changing with advances in medicine. Thus, our results are not directly transferable to other populations in different medical situations. However, we believe that increased medical dependency and needs for supportive care in cachectic patients might be important features of cancer cachexia that can be shared in different medical situations.

For optimal management of cancer cachexia, a multimodal treatment approach including medication, exercise, and nutritional intervention are reported to be essential [6, 32]. However, there are currently no pharmacotherapies that are specifically approved for the treatment of cancer cachexia. A number of investigational agents are now in clinical development, including ghrelin and ghrelin mimetics [10, 11], selective androgen receptor modulators [9], and anti-inflammatory agents [33]. In addition, there is limited evidence for non-pharmacological treatments including nutrition and exercise intervention for patients with advanced cancer [34]. Recently, Solheim TS et al. [12] reported the results of a randomized phase II study comparing a multimodal intervention (exercise, nutritional intervention, and anti-inflammatories) versus standard cancer care in patients with advanced NSCLC and pancreatic cancer (Pre-MENAC study, Clinical Trials Registry No. NCT01419145). They showed that the intervention was feasible and was associated with statistically significant weight gain. However, there was no significant improvement in muscle mass or physical activity. The MENAC study, a phase III randomized, open-label trial of this multimodal intervention plus standard care vs. standard care alone to prevent cachexia in advanced cancer patients undergoing chemotherapy, is now underway (Clinical Trials Registry No. NCT02330926).

Based on the results of our study, we are currently conducting a prospective multicenter feasibility study of early exercise and nutritional intervention for elderly patients with advanced NSCLC and pancreatic cancer in Japan (Clinical Trials Registry No. UMIN000023207). We hypothesize that early non-pharmacological supportive care might maintain physical function and hence reduce medical dependency and costs in elderly patients with advanced cancer at high risk for cachexia.

## Conclusion

Cachectic elderly patients with advanced NSCLC were more frequently disabled, required prolonged hospitalizations, and were associated with higher medical costs while receiving chemotherapy. Our results suggest that there is a potential need for early multimodal intervention with exercise and nutrition for elderly patients with advanced lung cancer to maintain functional independence and reduce medical dependency during chemotherapy. Further randomized control study is needed to determine the optimal treatment regimen for cancer cachexia and its impact on functional prognosis.

## Abbreviations

ADL: activity of daily living
BMI: body mass index
CT: computed tomography
DFS: disability-free survival
NSCLC: non-small cell lung cancer
OS: overall survival
